# Cajal Body Proteins Differentially Affect the Processing of Box C/D scaRNPs

**DOI:** 10.1371/journal.pone.0122348

**Published:** 2015-04-13

**Authors:** Isioma I. Enwerem, Guowei Wu, Yi Tao Yu, Michael D. Hebert

**Affiliations:** 1 Department of Biochemistry, The University of Mississippi Medical Center, Jackson, Mississippi 39216–4505, United States of America; 2 Department of Biochemistry and Biophysics, The University of Rochester Medical Center, Rochester, New York 14642, United States of America; CNRS UMR7275, FRANCE

## Abstract

Small nuclear ribonucleoproteins (snRNPs), which are required for pre-mRNA splicing, contain extensively modified snRNA. Small Cajal body-specific ribonucleoproteins (scaRNPs) mediate these modifications. It is unknown how the box C/D class of scaRNPs localizes to Cajal Bodies (CBs). The processing of box C/D scaRNA is also unclear. Here, we explore the processing of box C/D scaRNA 2 and 9 by coilin. We also broaden our investigation to include WRAP53 and SMN, which accumulate in CBs, play a role in RNP biogenesis and associate with coilin. These studies demonstrate that the processing of an ectopically expressed scaRNA2 is altered upon the reduction of coilin, WRAP53 or SMN, but the extent and direction of this change varies depending on the protein reduced. We also show that box C/D scaRNP activity is reduced in a cell line derived from coilin knockout mice. Collectively, the findings presented here further implicate coilin as being a direct participant in the formation of box C/D scaRNPs, and demonstrate that WRAP53 and SMN may also play a role, but the activity of these proteins is divergent to coilin.

## Introduction

Certain eukaryotic RNAs are subject to extensive post-transcriptional modification by pseudouridylation and 2′-*O*-methylation. Two examples are rRNA and small nuclear RNA (snRNA), and these modifications are necessary for optimal activity of ribosomes and spliceosomes [1,2]. Modification of rRNA, and RNA polymerase III-derived U6 snRNA, is directed by small nucleolar RNPs (snoRNPs) in the nucleolus [3]. There are two classes of snoRNPs, defined by the snoRNA component: box C/D, which catalyze 2′-*O*-methylation and box H/ACA which direct pseudouridylation. U1, U2, U4 and U5 snRNA, which are produced by RNA polymerase II, undergo an extensive maturation process (including steps in the cytoplasm) before transiently accumulating in the Cajal body (CB), a nuclear subdomain. These steps are under the egis of SMN, the survivor of motor neuron protein. Mutations in SMN are found in most patients with spinal muscular atrophy (SMA), the leading genetic cause of infant mortality [4–9]. In the nucleus, SMN is enriched within the CB and has been shown to interact with coilin, the CB marker protein [10,11]. It is speculated that SMN in the CB may take part in the recycling or regeneration of RNPs [12]. Upon the arrival of the nascent snRNP to the CB, the snRNA component is modified by small Cajal body-specific RNPs (scaRNPs). As with snoRNPs, there are different classes of scaRNPs defined by the scaRNA: box C/D (2′-*O*-methylation), box H/ACA (pseudouridylation) and mixed domain scaRNAs containing both box C/D and box H/ACA motifs. The different classes of scaRNAs recruit a set of core proteins, including fibrillarin (the methyltransferase for box C/D scaRNPs) and dyskerin (the pseudouridylase for box H/ACA scaRNPs). Complementary binding to the target region on the snRNA by the scaRNA of the scaRNP directs where the modification should take place on the snRNA.

Although there are many parallels between snoRNPs and scaRNPs, they are distinct given i) their location of action (nucleolus for snoRNPs and CB for scaRNPs) and ii) their RNA. Unlike snoRNAs, H/ACA scaRNAs contain a motif that targets these RNAs to the CB: the Cajal body localization box (CAB box) [13]. The protein WRAP53 (TCAB1/WDR79) binds the CAB box to recruit H/ACA scaRNAs to CBs [14,15]. A CAB box is also found in telomerase RNA (hTR), and WRAP53 interacts with this motif as well, resulting in the accumulation of hTR in CBs. Mutations in WRAP53 or hTR underlie some cases of dyskeratosis congenita, a genetic disorder caused by shortened telomeres [16]. In human, only box H/ACA or mixed domain scaRNAs contain CAB boxes [13,17]. In contrast, box C/D scaRNAs (2, 7, 9, 17) do not contain a CAB box in the appropriate context. It is therefore unclear how these RNAs are targeted to the CB and incorporated into scaRNPs. Human box C/D scaRNAs can be found in WRAP53 immunocomplexes, but only in significant amounts when cells are lysed in stringent conditions [15]. It is interesting to note that coilin, the CB marker protein, requires stringent conditions for its extraction [18], and these conditions were utilized in order to show an association of box C/D scaRNAs with WRAP53 [15]. However, a more recent study [19] was unable to repeat the findings of the 2009 study [15] and noted that changing the IP conditions did not increase the interaction of WRAP53 with box C/D scaRNAs. These authors conclude that WRAP53 binds approximately 20-fold less efficiently to box C/D scaRNPs compared to box H/ACA scaRNPs [19]. Moreover, there are some notable differences in scaRNP biogenesis across species. For example, human box C/D scaRNAs lack a consensus CAB motif to which WRAP53 binds. A CAB-like motif, however, is present in *D*. *melanogaster* box C/D scaRNAs and the fly homologue of WRAP53 can be crosslinked to this sequence [15]. In contrast, human WRAP53 fails to crosslink with *Drosophila* C/D CAB-like box-containing stemloops, which suggests that, in human, another protein binds to human C/D scaRNAs and targets these to CBs. Based on these findings, we hypothesized that WRAP53 indirectly interacts with box C/D scaRNAs and another *trans* factor besides WRAP53 is directly responsible for the localization of these scaRNAs to the CB. Given that coilin can form a complex with WRAP53 [20] and highly associates with box C/D scaRNAs 2 and 9 [21], we further hypothesized that coilin is this other *trans* factor. Its is interesting to note that the common characteristics shared between scaRNA 2 and 9 is that they are box C/D type scaRNAs that are subjected to processing [22]: For scaRNA2, mgU2-61 is generated while two guide RNAs are processed from scaRNA9: mgU2-19 and mgU2-30. Our previous work has shown that coilin has RNase activity [23] and can process scaRNA 2 and 9 in vitro [21], bolstering our belief that the CB marker protein directly takes part in scaRNP biogenesis.

In order to more thoroughly clarify box C/D scaRNP biogenesis, with particular emphasis on determining the impact that CB-enriched proteins have on this process, we examined SMN, WRAP53 and coilin in scaRNA2 processing assays. We also explored the in vitro processing of scaRNA 2 and 9 by coilin and examined box C/D scaRNP activity in a coilin knockout mouse embryonic fibroblast (MEF) cell line. The results of these experiments provide additional evidence supporting a direct role for coilin in scaRNP biogenesis. The studies shown here also demonstrate that, although WRAP53, SMN and coilin are all enriched in the CB, reduction of these proteins differentially impacts box C/D scaRNP biogenesis. These findings are discussed in terms of cell types that have CBs as well as cell types that lack this subnuclear domain but still require the machinery needed for pre-mRNA processing.

## Results

### ScaRNA9 is a better substrate for coilin processing than scaRNA2, and the GU region of scaRNA9 impacts coilin processing

**Fig 1:**
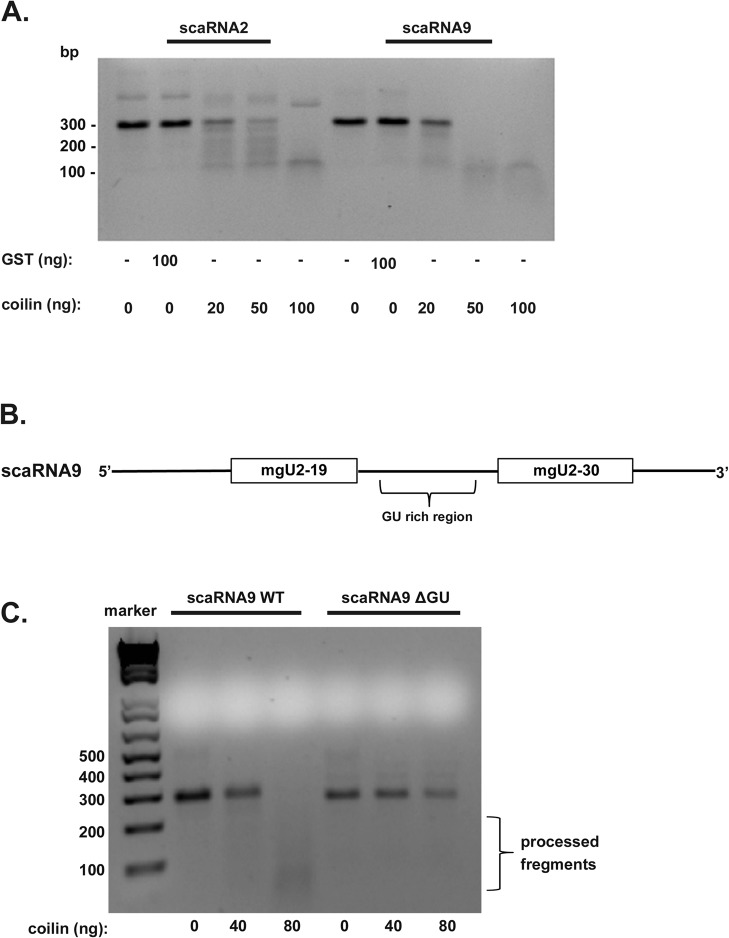
Coilin processing activity shows preference towards scaRNA9 compared to scaRNA2, and the GU region of scaRNA9 impacts coilin processing. (A) RNA processing assay comparing the processing of *in vitro* transcribed scaRNA2 and scaRNA9 by bacterially purified coilin. Equivalent amounts of RNA were incubated with no protein, GST (100 ng) or increasing amounts of pure, nucleic acid free coilin (20 ng, 50 ng and 100 ng). Products were resolved on a 2% agarose gel and detected by ethidium bromide staining. (B) Schematic showing full-length scaRNA9 (353 nucleotides in length), the mgU2-19 and mgU2-30 RNAs that are derived from the full-length RNA, and the GU-rich region that lies in between these smaller guide RNAs. (C) RNA processing assay comparing the processing of *in vitro* transcribed scaRNA9 and scaRNA9 with a deleted GU-rich region (scaRNA9 ΔGU) by purified coilin. Approximately equal amounts of RNA were incubated with no protein, or two amounts of pure, nucleic acid free coilin (40 ng and 80 ng). Products were resolved on a 2% agarose gel and detected by ethidium bromide staining.

Our previous work has shown that both scaRNA2 and scaRNA9 are substrates for coilin processing activity *in vitro* [21]. In the process of conducting this work, we observed that scaRNA9 appeared to be a better substrate for coilin processing activity compared to scaRNA2. To explore this further, we conducted RNA processing assays by incubating purified coilin or GST with approximately equal amounts of *in vitro* transcribed scaRNA2 or scaRNA9. The resulting products where then analyzed by agarose gel and stained with ethidium bromide (Fig 1). Negative control reactions lacking protein or containing 100 ng of purified GST do not show any processing/degradation. The addition of coilin, however, resulted in the processing of both scaRNA 2 and 9 in agreement with our previous results [21]. Interestingly, coilin amounts higher that 20 ng have a marked difference on the processing of scaRNA2 and scaRNA9 by coilin, with less full-length scaRNA9 detected compared to scaRNA2 (Fig 1A). These findings demonstrate that, in this experimental context, scaRNA9 is a better substrate for coilin processing than scaRNA2. To understand why this is so, we examined the sequence of scaRNA9 in more detail. Full-length scaRNA9 can be processed to generate two methylation guide RNAs, known as mgU2-19 and mgU2-30, that direct modification of U2 snRNA [22]. We have shown previously that the intervening region between these two guide RNAs is more susceptible to processing by coilin compared to other regions of this RNA [21]. This intervening region that is processed to produce the two guide RNAs contains a GU-rich dinucleotide repeat sequence (Fig 1B). Very interestingly, *Xenopus laevis* coilin has been shown to bind to G and U RNA homopolymers [24]. In an attempt to determine if coilin processing of scaRNA9 was via this GU-rich region, we deleted 42 nucleotides encompassing this area, replacing it with a 15 nucleotide non-GU-rich linker (scaRNA9 ΔGU). Indeed, we found that coilin processing of scaRNA9 was reduced when the GU region was deleted as compared to that observed with wild-type (Fig 1C). These results support the hypothesis that coilin is involved in the biogenesis of scaRNPs, with possible direct influence upon the processing of scaRNA 2 and 9.

### Coilin processing of scaRNA2 and scaRNA9 is non-random

**Fig 2:**
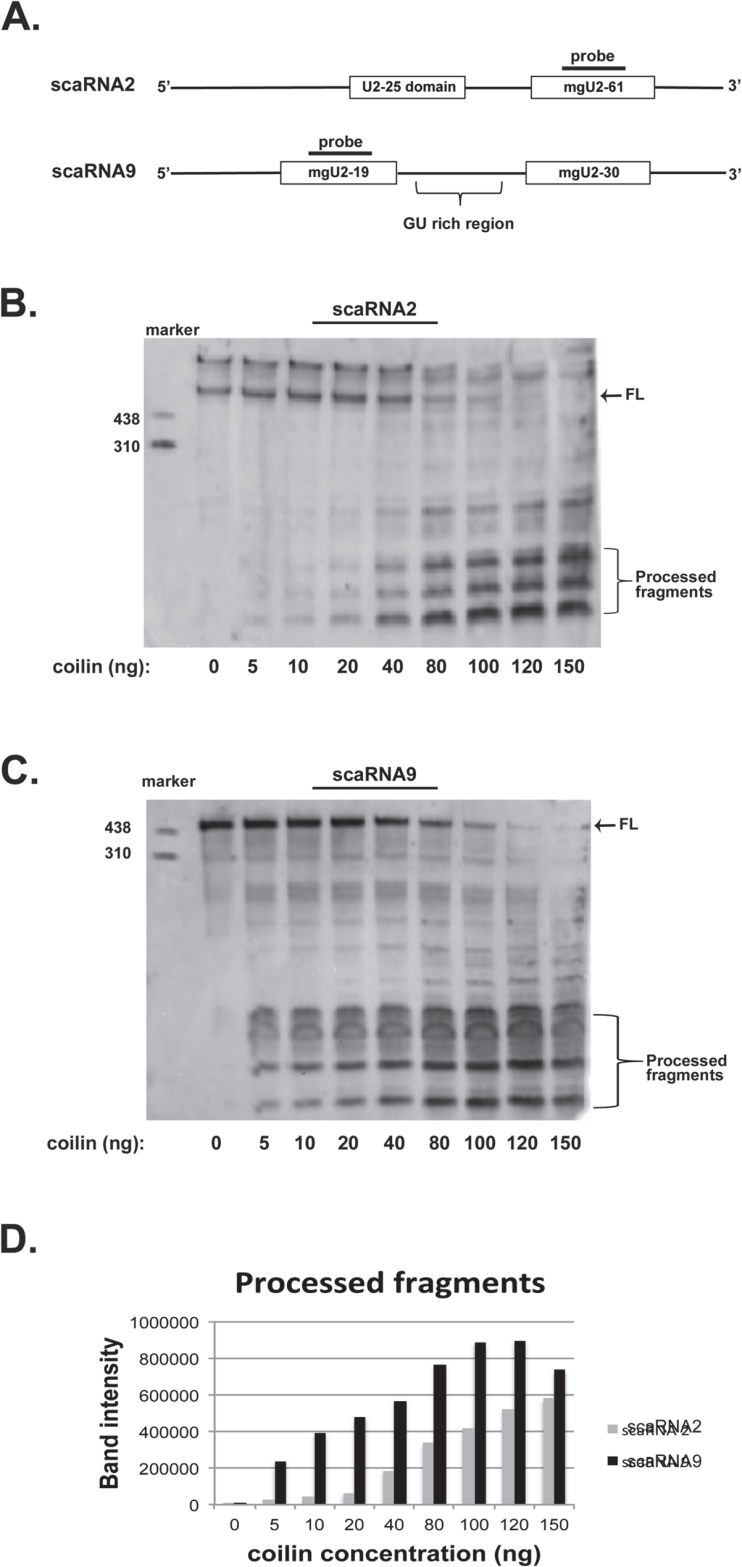
Coilin processing of scaRNA2 and scaRNA9 generates smaller, relatively stable, fragments. (A) Schematic showing full-length scaRNA2 and the guide RNA (mgU2-61) derived from this full-length RNA. Also shown are scaRNA9 and the two guide RNAs (mgU2-19 and mgU2-30) derived from this full-length RNA. The location of the probes used to detect scaRNA2 and scaRNA9, and their processed fragments, are shown. (B and C) Northern blot analysis of RNA processing experiments using *in vitro* transcribed scaRNA2 or scaRNA9 and increasing amounts of bacterially expressed purified coilin. After incubation, the reactions were subjected to acrylamide electrophoresis, Northern blotting and detection with the indicated probes. Full-length scaRNA 2 and 9 are indicated (FL) and processed fragments for both scaRNA2 and scaRNA9 are bracketed. The mobility of a DIG-labeled RNA marker (in nucleotides) is shown. (D) Quantification of the processed fragments from the scaRNA2 (B) and scaRNA9 (C) experiments.

To better assess the processed fragments generated upon incubation of scaRNA 2 and 9 with coilin, processing assays containing in vitro transcribed scaRNA2 or scaRNA9 with increasing amounts of purified coilin were run on polyacrylamide gels and Northern blotted. The blots were probed with DIG labeled oligos complementary to scaRNA2 or scaRNA9, as shown in Fig 2A. In agreement with the results shown in Fig 1, scaRNA9 is shown to be a better substrate for coilin processing compared to scaRNA2 in that processed smaller fragments can be observed in reactions containing only 5 ng of coilin with scaRNA9 (compare the 5 ng lane in Fig 2B to that in Fig 2C). Quantification of the processed fragment signals are shown in Fig 2D, and it can be easily observed that more processed scaRNA9 fragments are generated with increasing coilin amounts compared to that found for scaRNA2. Importantly, the presence of distinct bands indicates that coilin is not randomly degrading the scaRNAs, but is acting upon these RNAs in a specific manner. Moreover, the processed fragments are relatively resistant to additional coilin processing compared to full-length scaRNA 2 or 9 (for both Fig 2B and 2C, compare the amount of full-length signal to that for the processed fragments at 150 ng of coilin). The processed fragments were not detected in negative control reactions lacking coilin (0 ng lanes in Fig 2B and 2C).

### Reduction of CB proteins dysregulates ectopically expressed scaRNA2 processing

**Fig 3:**
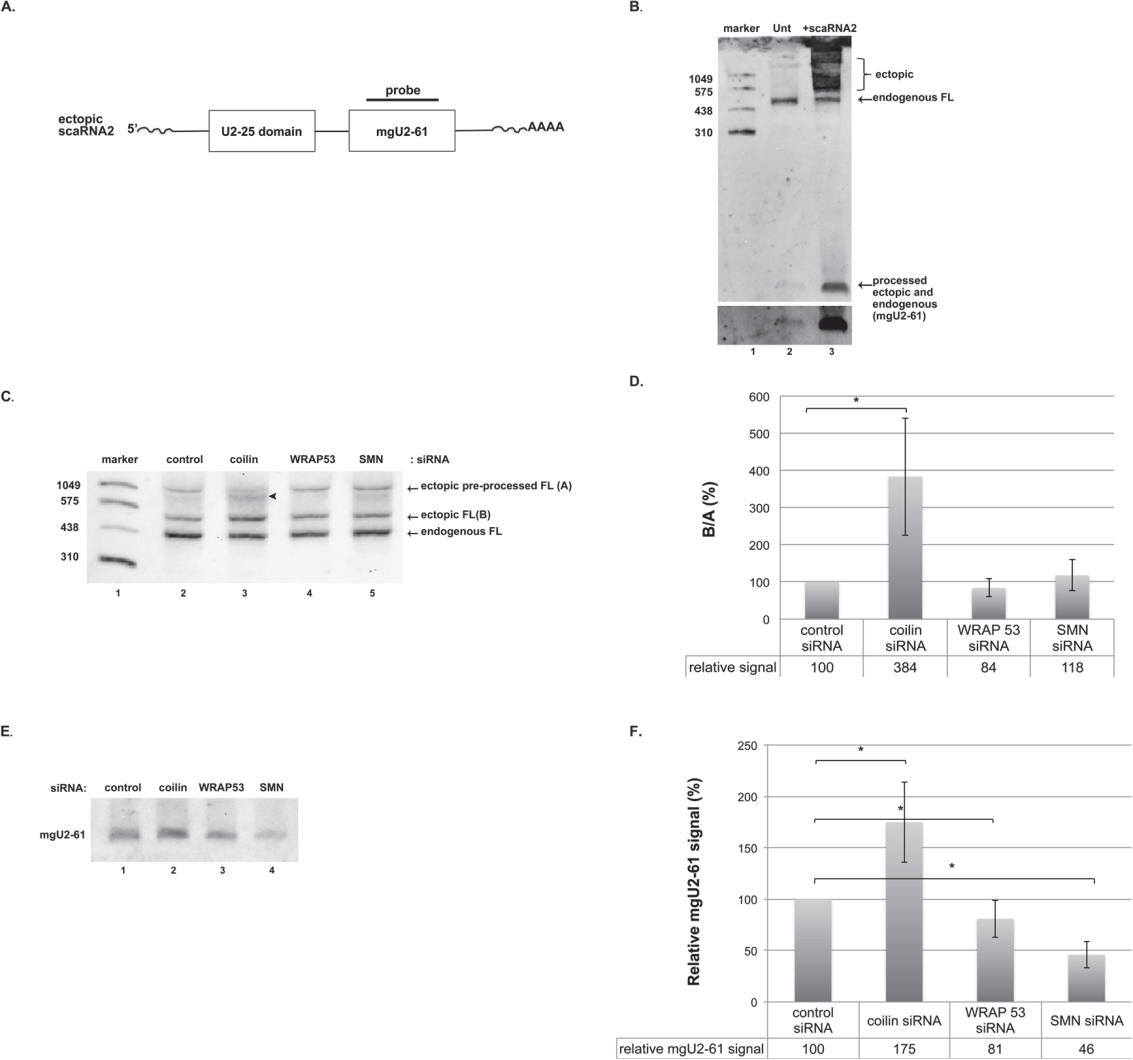
Reduction of coilin, WRAP53 or SMN dysregulates processing of ectopically expressed scaRNA2. (A) Schematic showing ectopic pre-processed full-length scaRNA2 (expressed from the pcDNA3.1+ vector) and the locations of the U2-25 domain and the processed guide RNA (mgU2-61). A poly A tail, which does not exist for endogenous scaRNA2, is indicated. The probe used for Northern blots is also shown. (B) Northern blot of HeLa RNA (20 μg/lane) from either non-transfected cells (lane 2) or cells transfected (24 hr) with pcDNA 3.1+ scaRNA2 (lane 3). The probe used is shown in (A) The lower panel shows a longer exposure of the region of the gel with mgU2-61 signal in order to better visualize the endogenous signal. (C) Northern blot of Hela RNA (10 μg/lane) from cells transfected with control (lane 2), coilin (lane 3), WRAP53 (lane 4) or SMN (lane 5) siRNA for 24 hrs then transfected with pcDNA 3.1+ scaRNA2 for 24 hrs. The probe used is shown in (A). Ectopic pre-processed full-length (denoted as A), ectopic processed full-length (denoted as B) and endogenous full-length scaRNA2 is shown. An arrowhead marks an intermediate species often seen in RNA isolated from coilin knockdown cells (lane 3). (D) Histogram quantifying the ratio between ectopic processed full-length and ectopic pre-processed full-length (B/A) from 8 different experimental sets. Standard deviation was used to include error bars. Students t test was used to determine statistical significance, indicated by “*” and corresponding to a p value less than 0.05. (E) Northern blot of Hela RNA (20 μg/lane) from cells transfected with control (lane 1), coilin (lane 2), WRAP53 (lane 3) or SMN (lane 4) siRNA for 24 hrs then transfected with pcDNA 3.1+ scaRNA2 for 24 hrs. The Northern blotting transfer conditions were optimized to retain the smaller, mgU2-61 guide RNA. The probe used is shown in (A). The majority of the signal is coming from ectopically expressed mgU2-61 guide RNA. (F) Histogram quantifying the mgU2-61 guide RNA from 8 different experimental sets. The mobility of a DIG-labeled RNA marker (in nucleotides) is shown. Standard deviation was used to include error bars. Students t test was used to determine statistical significance, indicated by “*” and corresponding to a p value less than 0.05.

Having gained important information about the processing of scaRNA 2 and 9 by coilin from our in vitro studies, we next wanted to examine if the processing of box C/D scaRNA was disrupted in vivo upon reduction of proteins enriched in the CB. For these studies, we examined the processing of scaRNA2 in the context of decreased levels of coilin, WRAPA53 or SMN. Previous work has shown that reduction of any of these proteins is sufficient to disrupt CBs [20,25,26]. Unlike many scaRNAs, scaRNA2 is transcribed from its own gene (SCARNA2). The full-length mature scaRNA2 is approximately 420 nts in length (known as mgU2-25/61), and this RNA can be subject to processing to yield a smaller (~ 80 nt) guide RNA, known as mgU2-61 [22]. Interestingly, transcription of the SCARNA2 gene continues past the 3' end of the mature (mgU2-25/61) RNA [27], suggesting that this RNA requires 3' processing. We have previously shown that the coilin RNase activity has specificity towards the 3' ends of precursor U2 snRNA and hTR [28]. Hence coilin may also take part in some aspect of scaRNA2 3' end processing. In order to easily visualize both the mature and smaller (mgU2-61) scaRNA2 RNAs, we cloned the cDNA encoding this RNA into an expression vector (Fig 3A). It should be noted that, while the ectopically expressed scaRNA2 contains a poly A tail (indicated in Fig 3A), endogenous scaRNA2 lacks this modification. HeLa cells were transfected with the pCDNA3.1+scaRNA2 vector, followed by RNA isolation and Northern blotting and probing. The location of the probe (DIG-labeled) used for the Northern blotting is shown in Fig 3A. As shown in Fig 3B, lane 2, we can detect endogenous full-length (mgU2-25/61, FL) and processed (mgU2-61) scaRNA2 in the RNA of untransfected cells using this probe. A band with the same size as mgU2-61 (~ 80 nts) is also detected in RNA isolated from cells transfected with pcDNA3.1+scaRNA2, but to a much greater extent compared to untransfected (Fig 3B, compare signals in lane 2 to that in lane 3 in both upper and lower panels). Hence the ectopically expressed scaRNA2 can be processed to generate the mgU2-61 guide RNA. Larger forms of ectopically expressed scaRNA2 are also detected as multiple bands, which we interpret as pre-processed (and polyadenylated), intermediate, and full-length (FL). We note FL ectopic scaRNA2 migrates slightly higher than endogenous FL scaRNA2, most likely because of the increased size of the ectopic RNA as a consequence of cloning and expression from a CMV promoter.

After verifying that the ectopically expressed scaRNA2 could be processed in vivo and detected by Northern blotting, we then transfected the scaRNA2 vector into cells with reduced levels of coilin, WRAP53 or SMN, followed by RNA isolation and Northern blotting. We examined the processing of the large (mgU2-25/61) and small (mgU2-61) fragments separately in order to optimize their Northern transfer. Representative gels are shown, as are histograms showing the quantification from 8 experimental repeats. For the processing of the large ectopic scaRNA2 fragment, we observed that coilin reduction significantly increases the relative amount of ectopic FL (indicated as B) compared to ectopic pre-processed (indicated as A; hence the B/A ratio) when compared to control, WRAP53 or SMN RNAi treatments (Fig 3C). Quantification demonstrates that coilin reduction increases the amount of the B/A ratio approximately 4-fold compared to control (Fig 3D). Additionally, RNA isolated from cells with reduced coilin and expressing ectopic scaRNA2 often have increases in the amount of what we define as an intermediate processed scaRNA2 species (Fig 3C, lane 3, arrowhead). Interestingly, the abundance of the small guide RNA (mgU2-61) is increased upon coilin knockdown but decreases when WRAP53 or SMN is reduced (Fig 3E, quantified in Fig 3F). These differences are statistically significant. We conclude from this analysis that ectopically expressed scaRNA2 processing is dysregulated when coilin, WRAP53 or SMN levels are altered. We could not detect changes in the processing of endogenous scaRNA2 upon reduction of coilin, WRAP53 or SMN (data not shown), possibly indicating a compensatory mechanism by the cell in response to disrupted CBs.

### Coilin reduction does not change the localization of mgU2-61

**Fig 4:**
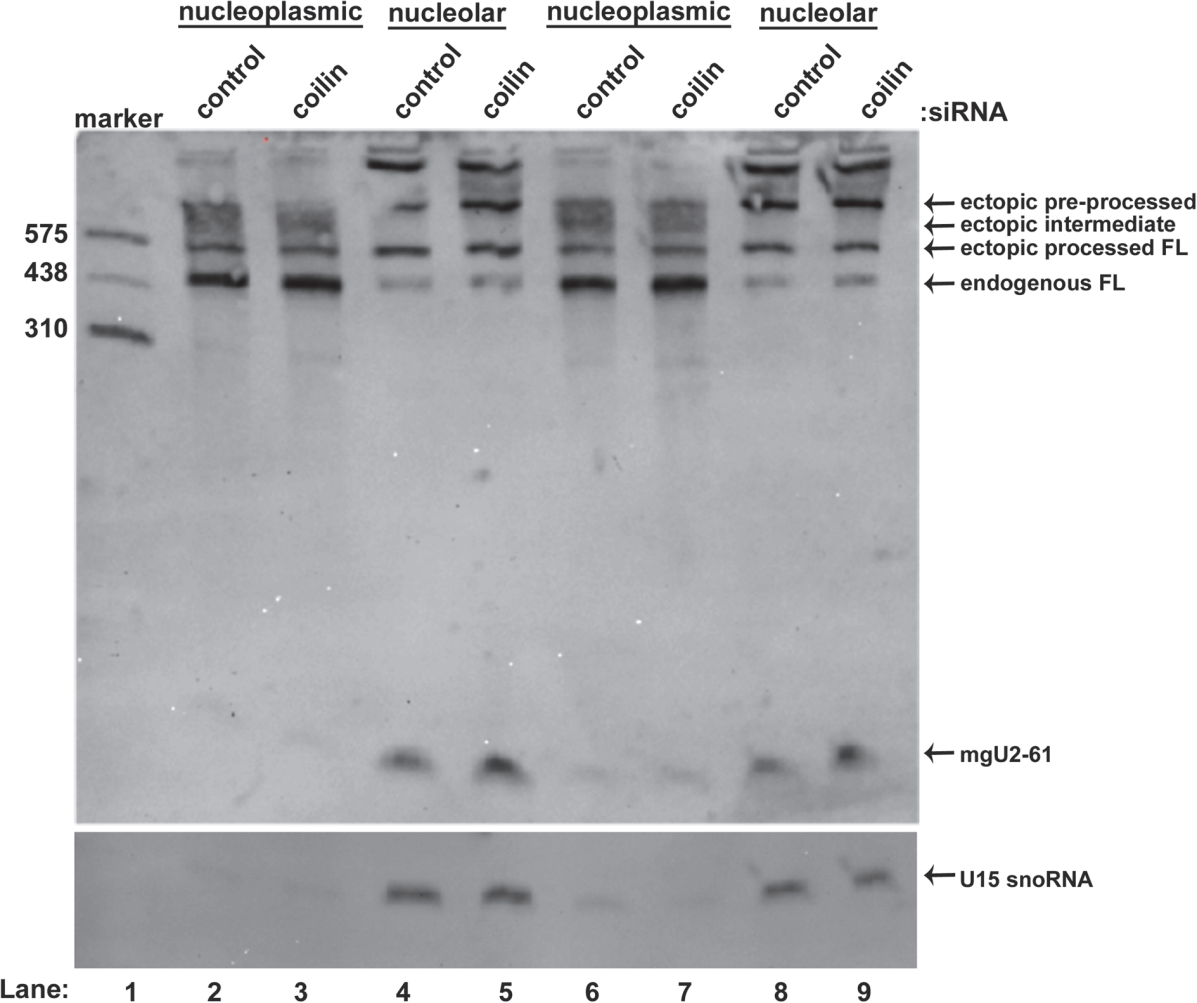
The scaRNA2-derived mgU2-61 guide RNA is enriched in the nucleolar fraction upon coilin reduction. HeLa cells transfected with control or coilin siRNA were fractionated to produce nucleoplasmic and nucleolar fractions. 5 μg/lane of RNA from each fraction was run on a 6% polyacrylamide gel and Northern blotted, followed by detection with a scaRNA2 probe (shown in Fig 3). Ectopic pre-processed, intermediate-processed and processed full-length species are indicated, as is the smaller guide RNA, mgU2-61. The blot was reprobed with an U15 snoRNA probe to verify the fidelity of the nucleolar fractionation and demonstrate that an approximately equal amount of RNA was loaded (lower panel). The mobility of a DIG-labeled RNA marker (in nucleotides) is shown. Duplicate samples are shown in lanes 6–9.

Previous work has shown that FL scaRNA2 (mgU2-25/61) is enriched in the nucleoplasm (presumably in CBs) while the smaller guide RNA (mgU2-61) derived from mgU2-25/61 is enriched in the nucleolus [22]. Since we have shown that coilin, WRAP53 or SMN reduction alters the level of mgU2-61 (Fig 3E), we next wanted to assess if the localization of this guide RNA was likewise affected. To accomplish this, HeLa cells were transfected with control or coilin siRNA, followed by transfection with pcDNA3.1+scaRNA2. At harvest, nucleoplasmic and nucleolar fractions were obtained and RNA was isolated from these fractions and subject to Northern blotting (Fig 4). In agreement with the data shown in Fig 3, the amount of mgU2-61 was increased in RNA isolated from coilin knockdown cells compared to control siRNA treated cells. The nucleolar enrichment of mgU2-61, however, did not appear to be greatly affected upon coilin reduction. The blot was reprobed with an oligo complementary to the nucleolus-enriched U15 snoRNA (lower panel) in order to serve as a fractionation and loading control. Relative to the amount of the U15 snoRNA signal, mgU2-61 levels are 2.3-fold increased in coilin- vs control-siRNA treated cells. Interestingly, the amount of ectopic intermediate processed scaRNA2 is greatly reduced in the nucleolar fraction compared to that found in the nucleoplasmic fraction, suggesting that scaRNA2 processing takes place in the nucleoplasm. Also, endogenous full-length scaRNA2 (mgU2-25/61) is predominantly enriched in the nucleoplasmic fraction, as previously reported [22]. Interestingly, the overexpression and fractionation of ectopic scaRNA2 demonstrates that ectopic pre-processed and ectopic full-length scaRNA2 appears to be relatively enriched in the nucleolar fraction. Based on this observation, it is possible that the trafficking of scaRNA2 involves a nucleolar step. Alternatively, the overexpression of scaRNA2 may be disrupting the normal trafficking and localization route that is followed by endogenous scaRNA2.

### Co-expression of SMN and coilin in WI-38 increase nuclear foci

**Fig 5:**
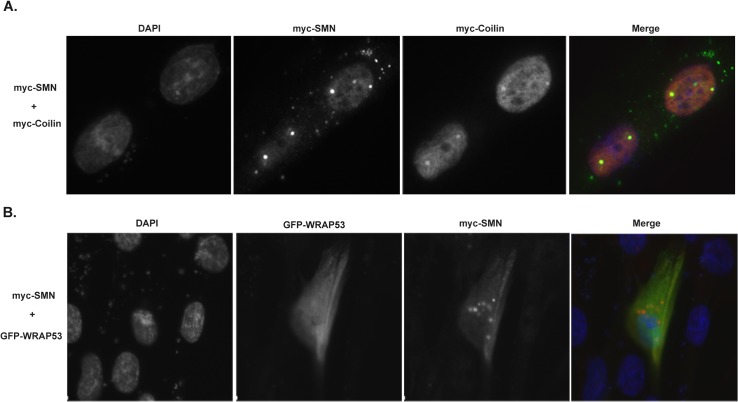
Co-expression of SMN and coilin in the WI-38 primary cell line promotes foci formation. (A) WI-38 cells were co-transfected with myc-tagged SMN and coilin, followed by fixation and detection of the expressed proteins using anti-SMN or anti-coilin antibodies and appropriate secondary antibodies. DAPI was used to stain the nucleus. In the merged image, the nucleus is blue, SMN is green and coilin signal is red. Foci with co-localized SMN and coilin are yellow. (B) WI-38 cells were co-transfected with myc-SMN and GFP-WRAP53, followed by fixation and detection of the expressed proteins. Anti-SMN antibody was used to detect SMN and the nucleus was stained with DAPI. In the merged image, SMN is red, GFP-WRAP53 is green and the nucleus is blue.

In addition to examining scaRNA processing in the transformed HeLa line, we were also interested in exploring scaRNP biogenesis in a primary cell line that lacks numerous CBs, such as WI-38. Compared to HeLa, WI-38 cells have been shown to have lower levels of SMN, coilin and WRAP53 protein [21] as well as few Cajal bodies [29]. Despite having a low percentage of cells with CBs, WI-38 cells still splice pre-mRNA and thus still require spliceosomal snRNA modification mediated by scaRNPs. These modifications in WI-38 presumably take place in the nucleoplasm. In HeLa, the majority of coilin (70%) is actually nucleoplasmic with only 30% being enriched within CBs [30]. This strongly suggests that coilin has activity that takes place in the nucleoplasm. This activity may or may not be separate from coilin function in the CB. Although expressed at lower levels in WI-38 compared to HeLa, coilin, SMN and WRAP53 protein are present in this primary cell line, implying that these proteins have activity in this cell line despite the dearth of CBs. It is in this cellular context that we next examined scaRNA2 processing. For these studies, we first examined if we could induce CB formation by overexpressing coilin, SMN or WRAP53. When tested individually, overexpression of SMN, coilin or WRAP53 failed to induce CBs. In contrast, co-expression of myc-SMN and myc-coilin resulted in the formation of nuclear foci that accumulated both SMN and coilin, consistent with CBs (Fig 5A). It should be noted that endogenous WRAP53 was not enriched in the foci formed in cells co-expressing myc-SMN and myc-coilin (data not shown). Co-expression of GFP-WRAP53 with myc-SMN or myc-coilin did not result in the formation of foci that included GFP-WRAP53. Indeed, overexpression of myc-SMN, which can lead to SMN foci in the cytoplasm, failed to recruit GFP-WRAP53 (Fig 5B). We conclude from these studies that co-expression of SMN and coilin is sufficient to form nuclear structures consistent with CBs in the WI-38 primary cell line.

### Co-expression of SMN and coilin in WI-38 alters processing of ectopically expressed scaRNA2

**Fig 6:**
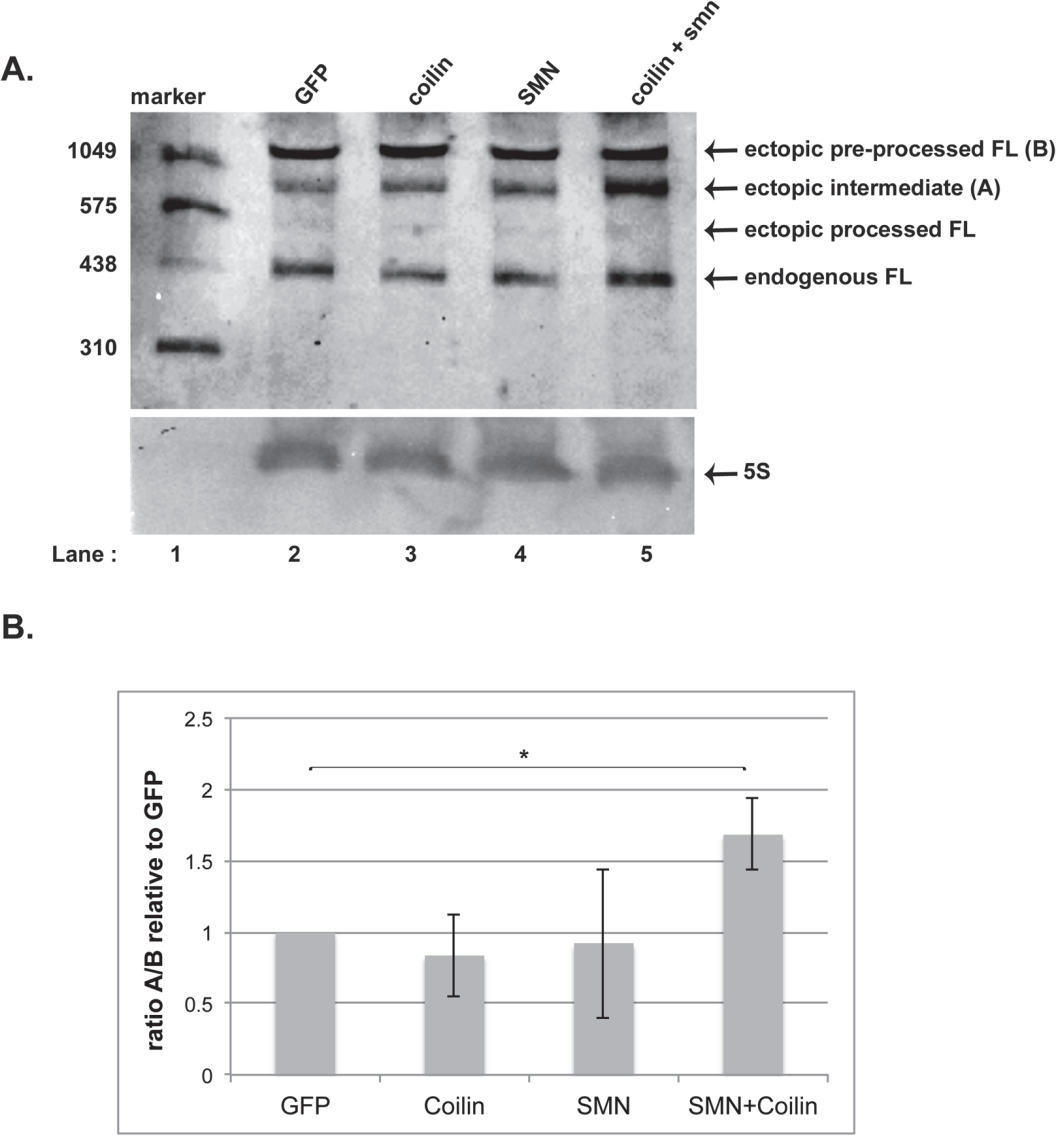
Co-expression of SMN and coilin increases processing of ectopically expressed scaRNA2 in WI-38 cells. (A) WI-38 cells were co-transfected with pCDNA3.1+scaRNA2 and empty GFP vector (lane 2), myc-coilin (lane 3), myc-SMN (lane 4) or myc-coilin + myc-SMN (lane 5) for 24 hrs followed by RNA isolation. RNA (10 μg/lane) was run on a 6% polyacrylamide gel, Northern blotted and detected using a DIG labeled oligo probe that detects scaRNA2 (shown in Fig 3). The positions of ectopic pre-processed full-length scaRNA2 (indicated as B), ectopic intermediate-processed scaRNA2 (denoted as A), ectopic processed full-length scaRNA2 and endogenous full-length scaRNA2 are shown. The blot was then probed for 5S ribosomal RNA (5S) to verify equivalent loading of RNA (lower panel). (B) Histogram showing quantification of the ectopic intermediate (denoted as A) to ectopic pre-processed FL (denoted as B) ratio for 3 experimental sets represented in panel A. The mobility of a DIG-labeled RNA marker (in nucleotides) is shown.Standard deviation was used to include error bars. Students t test was used to determine statistical significance, indicated by “*” and corresponding to a p value less than or equal to 0.05.

To test if ectopic scaRNA2 processing in WI-38 would be affected by the co-expression of myc-SMN and myc-coilin (which induce the formation of CB-like structures), we co-transfected pcDNA3.1+scaRNA2 with different plasmid DNAs. Before co-transfecting, we first isolated RNA from WI-38 cells transfected only with pcDNA3.1+scaRNA2 and subjected this RNA to Northern blotting with the probe shown in Fig 3A. We could not detect the small guide RNA, mgU2-61, in WI-38 cell RNA, even with ectopic scaRNA2 overexpression. Furthermore, the amount of ectopic processed FL scRNA2 was greatly reduced in WI-38 cells compared to that found in HeLa cells. This is evident in Fig 6A, lane 2, which shows Northern blotting of RNA isolated from WI-38 cells co-transfected with pcDNA3.1+scaRNA2 and empty GFP vector (as a control). Based on these findings, we hypothesized that the co-expression of myc-SMN and myc-coilin would increase the processing of ectopically expressed scaRNA2, resulting in the detection of the mgU2-61 guide RNA. To test this hypothesis, RNA was also isolated from cells co-transfected with pcDNA3.1+scaRNA2 plus myc-coilin (lane 3), myc-SMN (lane 4) or myc-SMN and myc-coilin (lane 5). In none of these situations could we detect the smaller mgU2-61 guide RNA. It is important to note that WI-38 cells are different from HeLa in terms of transformation status, cell type and developmental age. All these factors may contribute towards the biogenesis of scaRNPs. Since nucleoli in primary cells are typically smaller and fewer in number than those found in transformed cells, it is likely that the rRNA modification machinery in WI-38 cells is downregulated compared to that found in HeLa cells. A reduced level of mgU2-61 in WI-38 cells compared to that found in HeLa is consistent with this hypothesis. However, we did observe that the amount of intermediate processed ectopic scaRNA2 (indicated as A in Fig 6A) was increased relative to the ectopic pre-processed species (indicated as B) in cells co-expressing SMN and coilin (quantified in Fig 6B). This blot was reprobed with an oligo complementary to 5S rRNA to verify that an equivalent amount of RNA was loaded in each well (Fig 6A, lower panel). These findings indicate that co-expression of SMN and coilin may promote processing of scaRNA2 and/or stabilizes the intermediate processed form.

### Coilin depletion reduces U2 snRNA 2'-O-methylation

**Fig 7:**
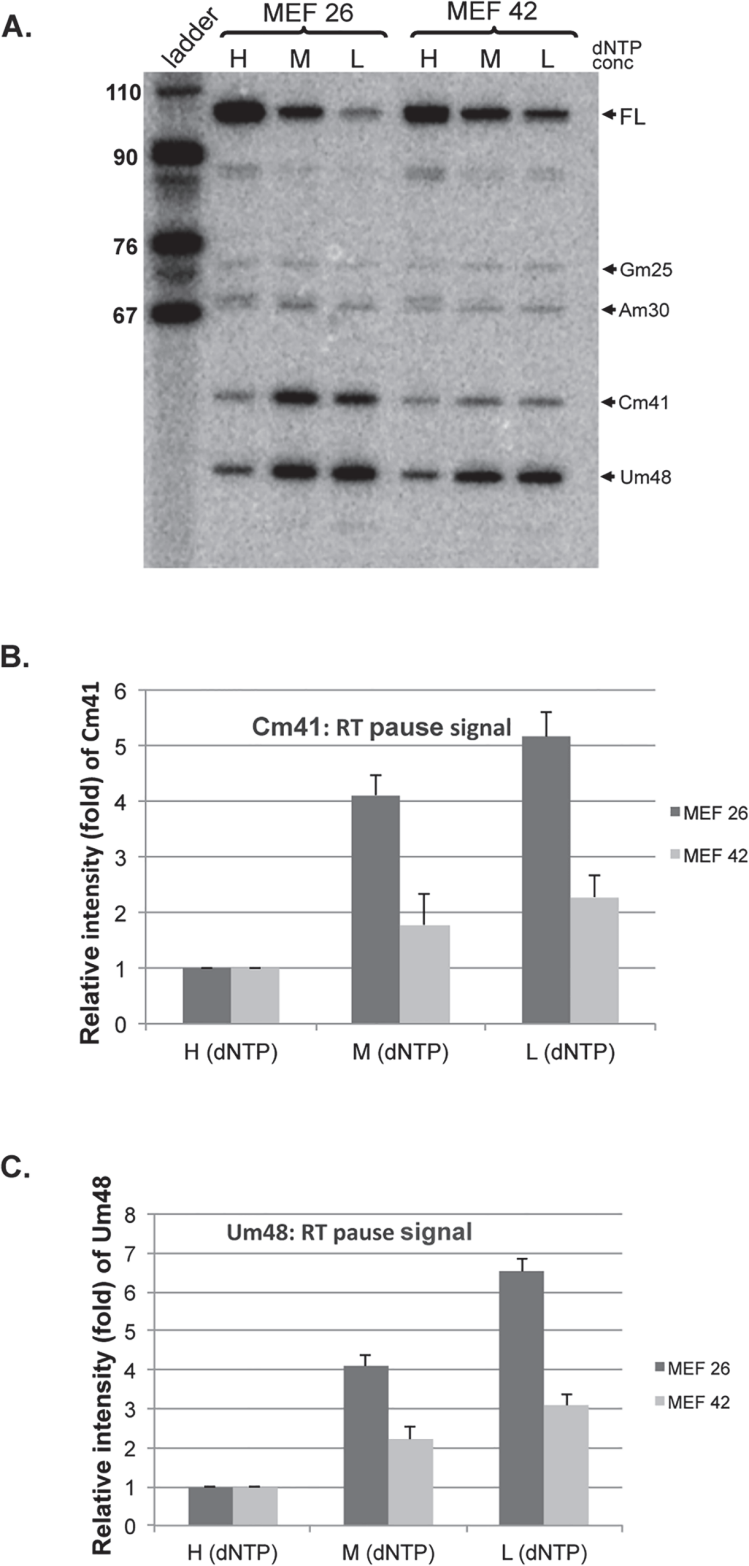
2′-*O*-methylation of U2 snRNA is reduced in coilin knockout MEF cells. **(A)** Primer extension experiments were done to map the 2’-*O*-methylation of individually modified nucleotides in U2 snRNA using equal amounts of RNA isolated from MEF 26 (coilin wild-type) and MEF 42 (coilin knock-out) cell lines. High, medium and low (H,M and L) deoxynucleoside triphosphate concentrations were used. The resulting pause signals corresponding to 2′-*O*-methylated residues in mouse U2 snRNA are indicated by each arrow. (B) Relative levels/intensities of Cm41 and Um48 in MEF26 and MEF 42 cell lines were calculated. For each lane, the percentage of the Cm41 and Um48 signals was calculated. The percentage values of Cm41 and Um48 of the medium and low dNTP lanes were then normalized to that of the high dNTP lanes. The ladder is shown in nucleotides.

The data shown above support the idea that proteins enriched within the CB, such as WRAP53, SMN and coilin, take part in box C/D scaRNP biogenesis. If coilin is directly involved in box C/D scaRNP biogenesis, as indicated by the data shown above, alterations in snRNA modification should be detected in cells with depleted coilin. A previous report using a coilin knockout mouse embryonic fibroblast line (MEF42) stated that no changes in U5 or U2 snRNA modification were found compared to the WT cell line (MEF26) [31]. The data for U5 snRNA was shown in the paper, and there are no obvious changes. However, the data for U2 snRNA was not shown. Considering that coilin highly associates with box C/D scaRNAs (scaRNA 2 and 9) [21] that are responsible for the 2'-*O*-methylation of U2 snRNA, we re-evaluated these modifications in MEF42 cells. RNA isolated from MEF26 and MEF42 cells was subjected to phenol:chloroform:isoamyl alcohol (25:24:1) extraction and ethanol precipitation to obtain high purity RNA suitable for analysis using a primer extension method. As shown in Fig 7, we indeed observe a reduction in the 2'-*O*-methylation of specific U2 snRNA residues (C41 and U48) upon coilin depletion. This is an important result because it clearly demonstrates that vertebrate coilin is involved in RNP biogenesis, most likely in the formation of scaRNP 2 and scaRNP 9 particles.

We also examined the processing of ectopically expressed scaRNA2 in MEF cells (data not shown). We were able to detect full-length ectopic (human) scaRNA2 in both MEF26 and MEF42 cells. However, the overall signal for the ectopic scaRNA2 (pre-processed, intermediate, and full-length) was lower in MEF42 compared to MEF26. Furthermore, while we were able to detect a slight signal for mgU2-61 when we overexpressed scaRNA2 in MEF26 cells, we were unable to detect a signal for mgU2-61 in the MEF42 cell line. It appears that there is less efficient expression of our scaRNA2 construct in MEF42 cells compared to MEF26. We have previously shown that MEF42 proliferates more slowly and has a reduced transcription rate compared to MEF26 [32], and these findings may account for the observed paucity of mgU2-61 signal in MEF42.

## Discussion

### Differential effect of CB proteins on box C/D scaRNA processing

The data presented here support the hypothesis that SMN, WRAP53 and coilin contribute to human scaRNP formation. Our initial analysis did not show any obvious change in the processing of endogenous scaRNA2 upon SMN, WRAP53 or coilin reduction. In order to overcome putative compensatory mechanisms that may help regulate the level and processing of endogenous scaRNA2 in response to protein knockdown, we next challenged cells 24 hrs after siRNA treatment with ectopically expressed scaRNA2. Cells were then harvested 24 hrs after transfection with the scaRNA2 DNA construct (resulting in 48 hr siRNA treatment and 24 hr ectopic scaRNA2 expression). Using these experimental conditions, we were able to identify alterations in the processing of ectopic scaRNA2 when SMN, WRAP53 or coilin levels were reduced. Very importantly, the reduction of SMN, WRAP53 and coilin all result in the disruption of CBs [20,25,26], yet there are differences in how ectopic scaRNA2 processing is altered in these conditions. Specifically, WRAP53 and SMN reduction decrease the amount of small guide RNA mgU2-61 detected, but increases of this guide RNA are seen with coilin reduction (Fig 3E and 3F). Fractionation studies (Fig 4) reveal that mgU2-61 is still predominantly nucleolar in the coilin knockdown condition, thereby suggesting that the disrupted processing of scaRNA2, and not the mis-targeting of this RNA, is responsible for the changes observed upon reduction of SMN, WRAP53 or coilin. The fractionation data also clearly show that there is more intermediate processed ectopic scaRNA2 in the nucleoplasmic fractions compared to the nucleolar fractions (Fig 4), indicating that the processing of scaRNA2 takes place in the nucleoplasm (or CB). We further note that coilin reduction increases the relative amount of processed ectopic scaRNA2 compared to control, SMN or WRAP53 RNAi treatments (Fig 3C and 3D). These findings argue for the idea that it is not the CB itself, but the factors that reside in the CB, that impact box C/D scaRNA processing. This concept is especially valid when considering that some cell types (WI-38) lack or have few CBs, but still express WRAP53, SMN and coilin, and still require scaRNPs to modify snRNA. In cells with an increased demand for scaRNPs, however, it is likely that CBs facilitate their formation. In support of this hypothesis, we have shown that overexpression of SMN and coilin in WI-38 cells increases the percent of cells with CB-like structures (Fig 5). This increase correlates with an increase in the amount of intermediate processed scaRNA2 (Fig 6), suggesting that ectopically expressed pre-processed scaRNA2 is being subject to more processing when a CB-like structure is present. Alternatively, the presence of higher levels of SMN and coilin in the WI-38 background may stabilize the intermediate form of ectopic scaRNA2. One goal for our studies is to recapitulate the processing of scaRNA2 as found for HeLa cells in the WI-38 background. In so doing, we should be able to better understand how box C/D scaRNP biogenesis takes place in both transformed and primary cells. Is it important to note that WI-38 cells are very different than HeLa cells not only in terms of cell type and transformation status but also in developmental age: HeLa is adult whereas WI-38 is embryonic. Studies have also shown that there is a marked difference in nuclear organization in embryonic cells compared to adult cells [33], so this is another variable that needs to be considered when formulating the mechanisms by which box C/D scaRNPs are generated.

### Coilin: A box C/D scaRNP assembly factor?

In this work we examined the impact of SMN, WRAP53 and coilin on the processing of an ectopically expressed box C/D scaRNA. Since snRNP, telomerase and scaRNP biogenesis are similar in that they require the assembly of proteins onto a non-coding RNA, and SMN associates with factors required for the formation of each class of RNPs, it is logical to conclude that SMN contributes to telomerase and scaRNP biogenesis in an equally important manner as it does for snRNP biogenesis. This idea is further strengthened when considering that SMN directly interacts with fibrillarin [34], the methyltransferase component of box C/D scaRNPs. Regarding WRAP53, previous work has shown that this protein interacts with the CAB box found in box H/ACA scaRNAs [14,15], including telomerase RNA (hTR), clearly indicating that this protein directly participates in scaRNP biogenesis. The role of WRAP53 in box C/D scaRNP formation is less clear, however, given that there is not a CAB motif present in box C/D scaRNAs [13]. Recent work has shown that WRAP53 binds approximately 20-fold less efficiently to box C/D scaRNPs compared to box H/ACA scaRNPs [19]. Remarkably, these same authors have observed that the G.U/U.G wobble stem of intron-encoded box C/D scaRNAs is required for their targeting to CBs and *in vivo* association with WRAP53 [19]. We note that this newly defined G.U/U.G *cis* element, the terminal stem loop (tSL) CB localization signal [19], is distinct from the GU dinucleotide repeats found most abundantly in scaRNA9 but also present in box C/D scaRNA 2 and 7. It was initially thought that these GU repeats could base pair with CA repeats that are abundant in many precursor and mature mRNAs [22]. More recent work, however, has noted that these GU repeats may serve as a CB localization signal [19] [35], and our work supports this hypothesis. Specifically, the high association of coilin with scaRNA 2 and 9 [21], which lack a terminal stem loop CB localization signal but contain GU repeats, indicates that coilin association with the GU dinucleotide repeats accounts in part for the enrichment of scaRNP2 and scaRNP9 in CBs. Coilin also associates with the independently transcribed scaRNA17 [21], which does not have a terminal stem loop CB localization signal or GU repeats, providing a possible mechanism by which scaRNP17 accumulates in CBs. By binding to different elements present in box C/D scaRNAs, coilin and WRAP53 ensure that the full contingent of scaRNPs are enriched in CBs. In cells without CBs, the interaction between coilin and WRAP53 would also be expected to faciliate scaRNP biogenesis.

Since coilin’s identification as the CB marker protein in 1991 [36], numerous studies have shown the importance of this protein on CB formation and composition [37]. Far fewer studies, however, have sought to understand the function of this protein in the nucleoplasm or in cell types that lack CBs. We have found that coilin is unlikely to simply be a building block for CBs. Instead, our results indicate that coilin may participate more directly in activities within the CB and nucleus than previously believed. Some of the important findings from these works are that coilin can bind nucleic acid (RNA and DNA) and has RNase activity [23,28,38]. Additional evidence linking coilin to RNP biogenesis comes from our comprehensive analysis of RNAs associated with coilin, which showed that box C/D scaRNA 2 and 9 were the most enriched (81-fold and 77-fold, respectively) RNAs present within the coilin complex [21]. Along with scaRNAs, we also detected in this previous work that snoRNAs were associated with coilin [21]. The association of coilin with scaRNAs and snoRNAs has recently been confirmed using a C-terminal GFP fusion of coilin (coilin-GFP) and UV crosslinking/ immunoprecipitation (iCLIP) to identify coilin interactions [39]. In addition to coilin association with scaRNAs, we have also shown previously that scaRNA 2 and 9 are in vitro substrates for coilin processing [21], and have extended these findings here. Namely, we have shown that coilin processing activity is preferential towards scaRNA9 relative to scaRNA2, and the GU-rich dinucleotide repeat region in scaRNA9 impacts coilin processing (Fig 1). Using a PCR based technique, our previous work indicated that the GU-rich region in scaRNA9 could be the target of coilin processing [21], and the work shown here supports this belief. Still additional evidence in support of a direct role for coilin in box C/D scaRNP formation comes from our processing assays that were subject to Northern blotting (Fig 2). The presence of discrete banding, instead of a smear, allows us to conclude that there is specificity in coilin’s processing of both RNAs. Furthermore, the amount of coilin required to generate smaller processed fragments was less when using scaRNA9 as a substrate compared to scaRNA2, again supporting the finding that scaRNA9 is a better coilin substrate than scaRNA2. Current studies are underway to examine the impact of coilin on scaRNA9 processing and localization in vivo. Our final result supporting a direct role for coilin in box C/D scaRNP biogenesis is shown in Fig 7, and shows that 2′-*O*-methylation of some U2 snRNA residues is reduced in coilin knockout MEF cells compared to wild-type.

**Fig 8:**
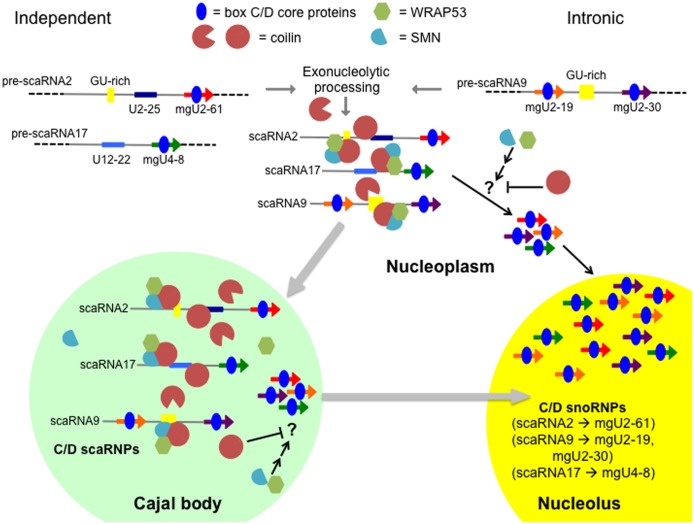
Model of box C/D scaRNP 2, 9 and 17 biogenesis. Box C/D core proteins (blue) are thought to bind the scaRNAs after transcription from independent genes (scaRNA2 and scaRNA17) or a host gene (for scaRNA9). For scaRNA9, the intron lariat is debranched for subsequent exonucleolytic processing to generate the mature scaRNA9. Exonucleolytic processing is also required to produce full-length scaRNA2 and 17 from longer precursor forms (dashed lines). Yellow boxes denote the GU dinucleotide repeats present in scaRNA2 and 9. Also shown are the smaller, nucleolus-enriched guide RNAs derived from scaRNA2 (mgU2-61, red), scaRNA9 (mgU2-19, orange and mgU2-30, purple) and scaRNA17 (mgU4-8, green). In cells without CBs (right pathway), these longer scaRNAs are processed in the nucleoplasm to generate the smaller guide RNAs that accumulate in the nucleolus as snoRNPs. In cells with CBs (left pathway, gray arrow to CB), full-length scaRNP 2, 9 and 17 accumulate in CBs, while the smaller guide RNA derived from these longer forms accumulate in the nucleolus. The mechanism(s) by which the cell governs the ratio of full-length scaRNA to smaller guide RNA is unknown (denoted as “?” in figure). Based on the data presented here, we believe that coilin, WRAP53 and SMN all participate at regulating the flux of scaRNA 2, 9 and 17 distribution and processing. Since coilin can both bind and process scaRNAs, it is possible that this protein contributes to the initial processing events that generate mature full-length scaRNA from the precursor forms containing 5’ and 3’ extensions. Coilin may also take part in the processing steps giving rise to the nucleolus enriched guide RNAs, although our coilin knockdown data (Fig 3) does not support this idea. Alternatively, coilin interaction with WRAP53 and SMN may decrease its processing activities, thereby allowing for the accumulation of full-length scaRNA 2, 9 and 17 in CBs. It is likely that another unknown factor (denoted as ‘?”) also contributes to the generation of the small guide RNAs derived from scaRNA 2, 9 and 17. Based upon the work presented here, we expect that coilin will negatively regulate the activity of this factor but SMN and WRAP53 will promote its processing activity (inhibition and activation arrow in nucleoplasm and CB).

Collectively, the data shown here support the hypothesis that coilin is a box C/D scaRNP assembly factor. More studies will be needed to clarify the exact function of coilin in this process, but based on our previous results and the data presented here, we propose a possible model (Fig 8). Central to this model is the idea that coilin can both bind and process box C/D scaRNAs. In so doing, coilin may contribute to the initial processing of the scaRNAs from the pre-processed RNA to the mature, full-length form incorporated into a scaRNP. Coilin association with these scaRNAs likely also contributes to their accumulation in CBs. Furthermore, the binding of coilin to box C/D scaRNAs may regulate subsequent processing events leading to the production of the smaller, nucleolus enriched guide RNAs (such mgU2-61). These nucleolar guide RNAs in turn would be expected to take part in the modification of rRNA. In the absence of coilin, this model predicts that an increase in the amount of smaller guide RNAs would result, and this is what we have observed for ectopically expressed scaRNA2 (Figs 3 and 4). In other words, coilin reduction may push the equilibrium of scaRNA2 processing towards the formation of the nucleolus-enriched mgU2-61 guide RNA. The decrease in U2 snRNA methylation observed in coilin knockout cells (Fig 7) may be indicative of scaRNA2 processing that is titled towards the formation of the smaller guide RNA, resulting in a reduction of scaRNPs needed to appropriately and fully modify U2 snRNA. Regarding SMN and WRAP53, it is possible that the decrease in the amount of mgU2-61 observed upon reduction of SMN or WRAP53 (Fig 3) is due to increased coilin binding to full-length scaRNA2, thereby dysregulating the normal processing events which govern the production of this smaller guide RNA. If true, this suggests that coilin, WRAP53 and SMN all participate in regulating the ratio of full-length to smaller guide RNA for box C/D scaRNAs. Consequently, these three proteins may impact the production of scaRNPs that modify snRNAs in the CB (or nucleoplasm) and snoRNPs that modify rRNA in the nucleolus. It is expected that this regulatory function occurs in cells with or without CBs. Future studies will explore the role of SMN, WRAP53 and coilin on the processing of other box C/D scaRNAs, scaRNA9 and scaRNA17. Ideally, these studies should examine endogenous scaRNA9 and scaRNA17 to preclude any complications resulting from the use of overexpression constructs. Other future work will explore how the post-translational modification of WRAP53, SMN and coilin impact scaRNP biogenesis.

## Materials and Methods

### Cell lines, cell culture, plasmids and transfections

HeLa and WI-38 cells were from the American Type Culture Collection (Manassas, VA, USA). MEF26 and MEF42 lines [40] were from Greg Matera (University of North Carolina, Chapel Hill, NC, USA). Lines were cultured as previously described [41]. WI-38, MEF26 and MEF42 cells were transfected with plasmid DNA using Lipofectamine 2000 transfection reagent (Invitrogen, Carlsbad, CA, USA) following the manufacturer's protocol. The non-targeting, control siRNA [42] was obtained from Thermo Scientific (LaFayette, CO, USA). Coilin siRNA (N004645.12.4) [43], SMN siRNA (N000344.12.6) and WRAP53 siRNA (N001143990.12.2) were obtained from Integrated DNA Technology (Coralville, IA, USA). We have previously shown that these coilin and WRAP53 siRNAs work well in reducing respective protein levels [21]. S1 Fig shows that the SMN siRNA reduces SMN protein levels approximately 80% after 24 hrs transfection. SiRNAs were transfected into HeLa cells using Lipofectamine 2000 (Invitrogen, Carlsbad, CA, USA) according to the manufacturer's suggested protocol. DNA plasmids were transfected into HeLa cells using Fugene HD (Promega, Madison, Wisconsin, USA) according to the manufacturer’s protocol.

### Cloning and mutagenesis

Clones of scaRNA2 and scaRNA9 in pBluescript KS+ vector were described previously [21]. ScaRNA2 and scaRNA9 were cloned into pcDNA 3.1+ vector using standard methods. The scaRNA9 clone in both pcDNA 3.1+ and pBluescript KS+ was mutated by site directed mutagenesis using the Quikchange Site Directed Mutagenesis Kit (Agilent, Santa Clara, CA, USA) to produce scaRNA9 deleted (del) GU clones in both vectors. The GU-rich region of scaRNA9 is in between the mgU2-19 and mgU2-30 guide RNAs. ScaRNA9 del GU is a mutant of scaRNA9 where a 42 nucleotide GU-rich region was replaced with a 15 nucleotide linker lacking GUs (GTs) (underlined in primer sequence): 5’- ATCGTCGCAGGATCCACGCACATGTGTTTATAAAGATAACAGC-3’ (forward) and 5’-GGATCCTGCGACGATTATGCCCTTATTGTTTTACATTGTTTTATAGTTTTGC-3’ (reverse). All DNA constructs were sequence verified.

### *In vitro* transcription, RNA degradation/processing assays

ScaRNA2 and scaRNA9 clones in pBluescript KS were *in vitro* transcribed as previously described [21]. RNA processing assays were performed using *in vitro* transcribed scaRNA2 and scaRNA9 or scaRNA9 del GU. Transcripts were incubated at 37°C for 30 min with no protein, pure nucleic acid free coilin or purified GST, as indicated. Coilin was purified as previously described [28] with modifications mentioned in [21]. Reactions were subjected to agarose gel electrophoresis and stained with ethidium bromide.

### Northern blotting and probing

Hela cells transfected with control, SMN, WRAP53 or coilin siRNA for 24 hr were additionally transfected with scaRNA2 cloned in pcDNA 3.1+. The cells were harvested 24 hr after the DNA transfection and RNA was isolated using TRI Reagent (Molecular Research Center, Cincinnati, OH, USA). WI-38 cells were co-transfected with different combinations of GFP, myc-SMN, myc-coilin and pcDNA 3.1+ scaRNA2. WI-38 cells were harvested 24hrs after transfection followed by RNA isolation, again using TRI Reagent. 20 μg of each RNA isolated from HeLa cells and 10 μg of each RNA isolated from WI-38 cells was run on a 6% or 8% denaturing polyacrylamide gel in 1X TBE at 200V for 30–40 minutes. The gel was then washed in 200 ml 1x TBE for 10 minutes with gentle shaking and the RNA was transferred onto a positively charged nylon membrane using the DNA Stacks (Invitrogen, Carlsbad, CA, USA) and the iBlot Gel Transfer device (Life Technologies, Grant Island, NY, USA). Program P8 for 7 minutes was used for optimal transfer of the smaller, processed RNA or 13 minutes for optimal transfer of full-length scaRNA 2. After transfer, the blot was rinsed quickly in distilled water and allowed to dry. The RNA was then cross-linked to the membrane using a UV cross-linker (UVP, Upland, CA) at a setting of 120,000μJ/cm^2^. The blot was then placed in a hybridization bottle and pre-hybridized using 15 ml of Ultrahyb Ultrasensitive Hybridization buffer (Ambion Life Technologies, Grand Island, NY) per blot for 30 min at 37°C with rotation in a hybridization oven. The following DNA oligo probes were used to detect scaRNA 2, scaRNA 9 and 5S ribosomal RNA [44]: 5’-CTCGTCTATCTGATCAATTCATCACTTCT-3’ (scaRNA2), and 5’-GGGTGGTATGGCCGTAGAC-3’ (5S ribosomal RNA). The probes were labeled using the DIG Oligonucleotide Tailing Kit, 2^nd^ Generation (Roche, Indianapolis, IN, USA) according to the manufacturer’s protocol. After pre-hybridization, 22 μl of tailed probe was added to 20 ml of Ultrahyb Ultrasensitive Hybridization buffer to a final concentration of 4.5 nM. The blot was then hybridized overnight at 37°C with slow rotation in a hybridization oven. The blots were then washed according to a published protocol [45], using the DIG Wash and Block Buffer Set (Roche, Indianapolis, IN, USA), with a few modifications: Anti-DIG antibody was used at 1:10,000 and the DIG Wash buffer steps were cut down to two 15 minute washes. Blots were imaged using a Chemidoc imager (Bio-Rad, Hercules, CA).

### *In vitro* RNA degradation/processing followed by Northern blotting

Reactions were set up with *in vitro* transcribed scaRNA2, scaRNA9 and increasing amounts of pure nucleic acid free coilin (0 ng-150 ng) as stated above. The reactions were then run on a 6% denaturing polyacrylamide gel in 1X TBE at 200V for 30–40 minutes. Northern blotting was then conducted as mentioned previously. The following DNA oligo probes were used to detect scaRNA2 and scaRNA9: 5’-CTCGTCTATCTGATCAATTCATCACTTCT-3’ (scaRNA2), and 5’-GGAAAGACTTCTGATGCTCAGATTTGGCTA-3’ (scaRNA 9).

### Cell Fractionation and Northern blot analysis

HeLa cells were transfected with either control or coilin siRNA as described above. 24 hrs after siRNA transfection, cells were transfected with scaRNA 2-pcDNA 3.1+. Cells were then harvested 24hrs after DNA transfection. Cytoplasmic, nucleoplasmic and nucleolar fractions were obtained using a published protocol [46] and RNA was isolated from these fractions using TRI Reagent (Molecular Research Center, Cincinnati, OH, USA). 5 μg of RNA was run on a 6% denaturing polyacrylamide gel in 1X TBE at 200V for 30 minutes, Northern blotted and probed with DNA oligos to scaRNA 2 and U15 snoRNA. The U15 snoRNA probe used was previously described [22]: 5’-GAGAACATCCCCAAGTC-3’. Washes and imaging were done as mentioned above.

### Immunofluorescence

WI-38 cells were grown on glass slides (Thermo Fisher Scientific, Waltham, MA) and transfected using Lipofectamine 2000 (Invitrogen, Carlsbad, CA, USA) with combinations of myc-SMN, myc-coilin, GFP-WRAP53 or empty GFP vector. Cells were fixed with 4% paraformaldehyde in PBS for 10 mins, permeabilized in PBS containing 0.5% Triton for 5 min, followed by washing in PBS. Cells were then stained for SMN and coilin using anti-SMN mouse monoclonal antibodies (1:100) (BD Transduction Laboratories, San Jose, CA) and rabbit polyclonal anti-coilin H300 (1:200) (Santa Cruz Biotechnology, Santa Cruz, CA). DAPI staining was used to define the nucleus. Images were captured and processed as previously described [41].

### Primer extension assay to detect 2′-O-methylation in U2 snRNA

RNA was isolated from MEF26 (wild-type) and MEF42 (coilin knockout) cells. Additional phenol:chloroform:isoamyl alcohol (25:24:1) extraction and ethanol precipitation were performed to ensure high purity of RNA. 2 μg of purified RNA was subject to primer extension with high (1 mM), medium (0.01 mM) or low (0.005 mM) levels of dNTPs using a ^32^P labeled primer complementary to U2 snRNA (5' CCATTTAATATATTGTCCTCGG 3'), as previously described [47]. Primer-extension products were resolved on a 10% denaturing PAGE gel containing 8M urea.

## Supporting Information

S1 Fig SMN siRNA, and not control or coilin siRNA, reduces SMN protein levels. HeLa cells were transfected with siRNA. 24 hrs after transfection, cells were harvested and lysate was generated. Equal amounts of protein were resolved on SDS-PAGE, followed by Western transfer and detection of SMN using anti-SMN antibodies. Tubulin was then detected using anti-tubulin to verify equal protein loading.
(TIF)
