# Supplementary figures and images for: Cajal Body Proteins Differentially Affect the Processing of Box C/D scaRNPs
Source: PLoS One. 2015 Apr 13;10(4):e0122348. doi: 10.1371/journal.pone.0122348 (PMC4395269; doi:10.1371/journal.pone.0122348)

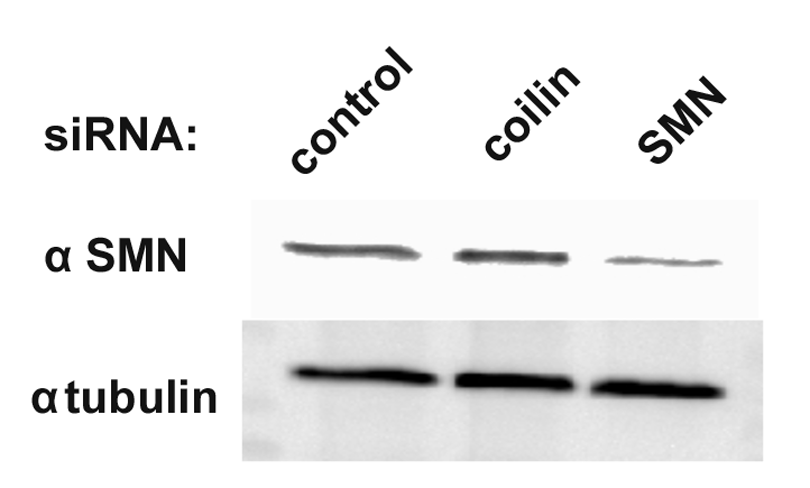

Supplement: S1 Fig — HeLa cells were transfected with siRNA. 24 hrs after transfection, cells were harvested and lysate was generated. Equal amounts of protein were resolved on SDS-PAGE, followed by Western transfer and detection of SMN using anti-SMN antibodies. Tubulin was then detected using anti-tubulin to verify equal protein loading. (TIF) [file pone.0122348.s001.tif]
